# High-Temperature Mechanical–Conductive Behaviors of Proton-Conducting Ceramic Electrolytes in Solid Oxide Fuel Cells

**DOI:** 10.3390/ma17194689

**Published:** 2024-09-24

**Authors:** Shimeng Kang, Penghui Yao, Zehua Pan, Yuhang Jing, Siyu Liu, Yexin Zhou, Jingyi Wang, Yan Gao, Yi Sun, Yongdan Li, Zheng Zhong

**Affiliations:** 1 School of Science, Harbin Institute of Technology, Shenzhen 518055, China; 22s058095@stu.hit.edu.cn (S.K.); zhouyexin@hit.edu.cn (Y.Z.); gaoyan@hit.edu.cn (Y.G.); zhongzheng@hit.edu.cn (Z.Z.); 2 Department of Chemical and Metallurgical Engineering, Aalto University, Kemistintie 1, FI-00076 Aalto, Finland; penghui.yao@aalto.fi (P.Y.); yongdan.li@aalto.fi (Y.L.); 3 Department of Astronautical Science and Mechanics, Harbin Institute of Technology, Harbin 150001, China; jingyh@hit.edu.cn (Y.J.); sunyi@hit.edu.cn (Y.S.); 4 Shenzhen Academy of Metrology & Quality Inspection, Shenzhen 518055, China; sliu018@outlook.com

**Keywords:** proton-conducting ceramics, proton diffusion, strain effect, elastic property, molecular dynamics

## Abstract

Proton-conducting solid oxide fuel cells (P-SOFCs) are widely studied for their lower working temperatures than oxygen-ion-conducting SOFCs (O-SOFCs). Due to the elevated preparation and operation temperatures varying from 500 °C to 1500 °C, high mechanical stresses can be developed in the electrolytes of SOFCs. The stresses will in turn impact the electrical conductivities, which is often omitted in current studies. In this work, the mechanical–conductive behaviors of Y-doped BaZrO_3_ (BZY) electrolytes for P-SOFCs under high temperatures are studied through molecular dynamics modeling. The Young’s moduli of BZY in fully hydrated and non-hydrated states are calculated with different Y-doping concentrations and at different temperatures. It is shown that Y doping, oxygen vacancies, and protonic point defects all lead to a decrease in the Young’s moduli of BZY at 773 K. The variations in the conductivities of BZY are then investigated by calculating the diffusion rates of protons in BZY at different triaxial, biaxial, and uniaxial strains from 673 K to 873 K. In all cases, the diffusion rate present a trend of first increasing and then decreasing from compression state to tension state. The variations in elementary affecting factors of proton diffusion, including hydroxide rotation, proton transfer, proton trapping, and proton distribution, are then analyzed in detail under different strains. It is concluded that the influences of strains on these factors collectively determine the changes in proton conductivity.

## 1. Introduction

Fuel cells are promising clean-energy technologies that convert chemical energy directly into electrical energy through electrochemical routes using hydrogen or other combustible gases as fuel and oxygen as the oxidant. Solid oxide fuel cells (SOFCs) use solid ceramics as the electrolyte and electrodes, operating at high temperatures and providing the highest efficiencies among all kinds of fuel cells [1,2,3,4,5]. For the traditional oxygen ion-conducting SOFCs (O-SOFCs), the commonly used electrolyte material is yttria-stabilized zirconia (YSZ). Because of the high activation energy of oxygen ion conduction in YSZ, O-SOFCs often operate at temperatures around 1073 K [6,7,8]. This leads to a long start-up time, fast degradation rate, and high cost. Many studies have focused on reducing the operating temperature of O-SOFCs by developing novel electrolyte materials, such as gadolinium-doped ceria (GDC) or La_0.9_Sr_0.1_Ga_0.8_Mg_0.2_O_3−*δ*_ (LSGM) [9,10,11,12]. However, the Ce^4+^ will reduce to Ce^3+^, leading to short circuit, and the mechanical properties of LSGM make it less suitable as the electrolyte in mass production. Recently, proton-conducting SOFCs (P-SOFCs) with proton-conducting ceramic electrolytes have attracted significant research interests. Owing to the low activation energy of proton transport, the operating temperatures of P-SOFCs can be as low as around 773 K [13,14,15], facilitating the practical deployment of SOFCs.

During the high-temperature sintering and cooling processes of SOFCs, through a variety of mechanisms, such as the mismatch of thermal expansion coefficients or a phase change, internal stresses can be developed in the electrolyte [16,17,18,19,20,21,22,23]. Yakabe et al. [18] analyzed the residual stresses in SOFCs using X-ray diffraction (XRD) combined with numerical simulations. It was revealed that the electrolyte was subjected to compressive stresses of up to 650 MPa at room temperature. Wang et al. [21] found that the principal stress of the electrolyte after inhomogeneous oxidation reached 1002 MPa. Later, they also found that the compressive stresses developed in the electrolyte became tensile stresses during the reduction and re-oxidation of the NiO through finite element simulations [22]. Kim et al. [23] analyzed the effects of the stress distributions under different contact conditions in a planar SOFC unit cell at 1073 K. The results showed that the electrolyte is the weakest component and has the maximum stress. When a perfectly bonded condition was applied, the maximum stress was calculated to be approximately 260 MPa, while when a static friction coefficient was set as 0.05, the maximum stress in the electrolyte was calculated to be around 25 MPa. Zhang et al. [24] investigated the effects of the mismatch in the material creep strength coefficient on the creep damage and failure probability of planar SOFCs by the finite element method. They found that the maximum equivalent creep strain and failure probability of SOFC were located in the outer corner of the sealant layer. From a microscale phase-field modeling perspective, Da et al. [20] analyzed the phase change in YSZ from the cubic to the tetragonal phase and pointed out that the phase change could cause von Mises stresses of up to 4 GPa, leading to higher failure probabilities, during long-term operation.

Most of the above studies mainly focused on the life-span and failure probabilities of the cell due the stress accumulation. However, the induced stress will also have an effect on the conductivity of electrolytes, which will in turn affect the discharging behavior of SOFCs and their long-term stability. For oxygen ion conductors like YSZ and GDC, a few experimental and simulation studies have demonstrated that compressive stress reduced the conductivity, while tensile stress increased the conductivity [25,26,27]. For example, the conductivity of Zr_0.82_Y_0.18_O_0.191_ was observed to decrease by 3% when the uniaxial compressive strain was 0.03% at 1073 K. The change in the conductivity was explained by the widened gap between cations under tensile stresses, since the transport of oxygen ions was through oxygen vacancies [28] and thus the widened gap reduced the activation energy of oxygen ion conduction.

For proton-conducting oxides, the transport mechanism of protons is different from the transport of oxygen ions. In perovskite oxides, mobile protonic defects can be produced by a hydration reaction, $H_{2}O+V_{O}^{••}+O_{O}^{\times }=2{\mathrm{OH}}_{O}^{\cdot }$ [29]. Proton transport typically follows a Grotthuss mechanism, which involves the rotation of hydroxide ions to make the proton closer to the next oxygen atom along with the transfer of protons from one oxide ion to another [13]. Studies on the effects of strain on the conductivities of proton conductors have not yet reached a consensus. For the effect of isotropic stress on the conductivity, Chen et al. [30,31] found that a compression strain would cause a decrease in the proton conductivity of Y-doped BaCeO_3_ (BCY), as well as an increase in the activation energy, measured by electrochemical impedance spectroscopy (EIS) at temperatures up to 398 K, ascribed to the smaller space available in the lattice. However, some other studies showed that a compression strain promotes proton conductivity. A first-principles molecular dynamics calculation performed by Fronzi et al. [32] revealed that isotropic compressive strain has a promoting effect on proton conduction in pure BaZrO_3_ at 1300 K. Similarly, Ottochian et al. [33] also found that compressive strain promotes proton conduction through reactive molecular dynamics at 1500 K to 2000 K, while tensile strain inhibits proton conduction. To elucidate the underlying mechanisms of the effect of strain, Niu et al. [34] investigated the influence of strain on the elementary steps of the diffusion of protons by analyzing the energy barrier through first-principles molecular dynamic simulation. They found that compression would hinder the rotation of the hydroxide ion while promoting the transfer of protons between oxygen atoms. For biaxial stress, the results of Ottochian et al. [35] predicted a parabolic trend of the diffusion coefficient as a function of strain and the maximum diffusivity occurred under compressive strain. Similarly, Fluri et al. [36] searched the conductivity variation in BZY thin films under biaxial stress from 700 K to 1300 K. The results also showed a parabolic trend of the diffusion coefficient upon the change in strain, as a result of the trade-off between the promotion of proton transfer and the inhibition of proton trapping.

Currently, there is a lack of studies that comprehensively consider the effects of strain on all the elementary factors affecting proton transport, including proton trapping, hydroxide rotation, proton transfer, and path preferences under anisotropic pressure, which could be the reason for the discrepancies in the study of proton conductivity variation upon applying stresses. In this study, the effect of stresses on the mechanical properties and proton transport was investigated by molecular dynamics. First, the change in Young’s modulus at different temperatures of BZY with different concentrations of Y doping was investigated. Then, the changes in the conductivity of BZY under uniaxial, biaxial, and isotropic strains at different temperatures were studied. The changes in hydroxide rotation, proton transfer, proton trapping, and proton distribution under anisotropic pressures were analyzed in detail to elucidate the mechanism of proton transport variation. Overall, these factors influence proton diffusion in the BZY collectively, in which isotropic compressive strain promoted proton transfer but inhibited hydroxide rotation and exacerbated proton trapping. For anisotropic strain, in addition to these factors, protons prefer to distribute on oxygen atoms with larger spatial volumes, which promotes proton diffusion in the tensile direction and inhibits proton diffusion in the compressive direction.

## 2. Calculation Methods

This study employed molecular dynamics (MD) for simulations. The potential was developed by Niu et al. [37] through machine-learning with data generated by first-principles calculation. All of the MD simulations were calculated by LAMMPS [38] (2 Aug 2023—Update 1). The subject of the study is 5%, 10%, 15%, and 20% Y-doped BaZrO_3_ (BZYx), denoted as BZY5, BZY10, BZY15, and BZY20, respectively. For all of the simulations, a 5 × 5 × 5 supercell was constructed containing 125 ABO_3_ perovskite formula units with a space point group of Pm$\bar{3}$m. The desired percentage of Zr atoms in the model were randomly replaced by Y atoms. For the non-hydrated BZY, a certain number of O atoms, equal to half of the number of Y atoms, were deleted in the model to keep electric neutrality. For the hydrated BZY, a certain number of protons, with the same number of Y atoms, were placed adjacent to random oxygen atoms. The initial distance between the proton and the bonded oxygen atom was 0.98 Å [39]. Upon relaxation, the distance ranges from 1.0 Å to 1.1 Å at temperatures between 300 K and 873 K.

The Young’s moduli of BZYx at different temperatures, 1 K, 300 K, 673 K, 773 K, and 873 K, were calculated by conducting compression simulations on the model under the NPT ensemble. The Young’s moduli were then derived from the slope of the stress–strain curve with a strain range of 1–5%.

In the investigation of the changes in proton conductivity, simulations were conducted under uniaxial, biaxial, and triaxial strain ranging from −4% to 4%. The simulation temperatures were between 673 K and 873 K. The conductivity of BZY was represented by the diffusion coefficient calculated from the mean squared displacement (MSD) according to Equation (1) [40]:

(1) $$\mathrm{MSD}\;\left(t\right)=1/N(\sum \langle {\left[r_{i}\left(t_{0}+t\right)-r_{i}\left(t_{0}\right)\right]}^{2}\rangle )=6Dt$$

where *N* represents the number of protons and *D* is the diffusion coefficient of the proton. The calculation of MSD was performed every 0.1 ps. Before the deformation, the model was relaxed under the NPT ensemble for 1 ns. After the deformation in the NPT ensemble, the NVT simulation was carried out for 1 ns to analyze the proton diffusion behavior, of which the first 200 ps was used for further equilibration. Due to the limited number of trajectories, segments of 200 ps were taken. The interval between the starting point of each segment was 50 ps. Thus, there were a total of 13 segments to average the MSD, with the last segment spanning from 800 ps to 1 ns. Trajectory files were exported in both unwrap and wrap formats. The unwrapped format was used to calculate the proton-diffusion coefficients, while the wrapped format was applied to analyze the conduction steps, such as hydroxide rotation and jumping.

**Figure 1:**
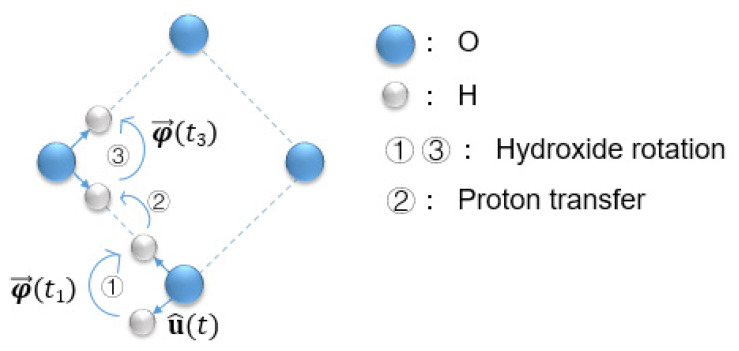
Illustration of the conduction of protons in a proton-conducting ceramic.

To further understand the underlying mechanisms for the effect of strain on the conductivity, the influence of strain on the main steps of proton diffusion was studied. As shown in Figure 1, proton diffusion involves two main steps: hydroxide rotation and proton transfer [36]. The angular motion of hydroxide ions was represented by the mean squared angle displacement (MSAD) [41] obtained by the following steps. First, the O ions closest to the protons in all frames were identified, and the unit vector pointing from each identified O atom to the adjacent H atom was denoted as $\hat{u}$. The expression $\hat{u}\left(t\right)\times \hat{u}\left(t+\Delta t\right)$ gives the direction of the instantaneous axis of rotation for a vector undergoing rotational displacement, where *t* represents the simulated time, with the magnitude of the angular displacement determined by $\left|\Delta \vec{\varphi }\left(t\right)|=\cos^{-1}(\hat{u}\left(t\right)\Delta \hat{u}\left(t+\Delta t\right)\right)$. Then, the total angular displacement was defined as

(2) $$\vec{\varphi }\left(t\right)=\int_{0}^{t}\Delta \vec{\varphi }\left(t\prime \right)dt\prime$$

The MSAD was then given by

(3) $$\mathrm{MSAD}=1/N\langle \Delta {{\vec{\varphi }}_{i}}^{2}\left(t\right)\rangle =1/N\langle {\left[{\vec{\varphi }}_{i}\left(t_{0}+t\right)-{\vec{\varphi }}_{i}\left(t_{0}\right)\right]}^{2}\rangle$$

Step 1 and Step 3: Protons approach the next oxygen atom through the rotation of the hydroxide ion; Step 2: Protons transfer to the next oxygen atom.

It is worth mentioning that the transfer of protons between different oxygen atoms leads to a change in the direction of $\vec{\varphi }\left(t\right)$, which is shown in Figure 1. The direction of $\vec{\varphi }\left(t_{1}\right)$ is opposite to $\vec{\varphi }\left(t_{3}\right)$, which means that $\vec{\varphi }\left(t\right)$ on different O atoms cannot be directly added together. Therefore, in this work, every time a proton jumped to another oxygen atom, the calculation of $\vec{\varphi }\left(t\right)$ in Equation (2) restarted at *t* = 0 and the MSAD result was added onto the previous result. That is, when the ***i***th proton made the *n*th rotation at a certain time point, *t_n_*, the total square angular displacement ($\mathrm{SAD}\left(t_{n}+\Delta t\right)$) for the proton was

(4) $$\mathrm{SAD}\left(t_{n}+\Delta t\right)={\left[{\vec{\varphi }}_{i}\left(t_{n}+\Delta t\right)-{\vec{\varphi }}_{i}\left(t_{n}\right)\right]}^{2}+\mathrm{SAD}\left(t_{n}\right)$$

The proton transfer was identified by the index of the nearest oxygen ion to the proton. When the index changed, proton transfer took place. In this way, the number of proton transfers was counted. Additionally, although periodic boundary conditions are applied in the simulation, the trajectory file has boundaries. When a proton approaches the edge of the boundary, the nearest oxygen atom to the proton may change due to crossing the boundary. This change is not necessarily due to the proton forming a bond with another oxygen atom. Therefore, to avoid the false appearance of multiple jumps caused by the proton repeatedly crossing the boundary, proton transfers are only counted when the distance between the oxygen atoms before and after the transfer is less than 6 Å.

## 3. Results and Discussion

### 3.1. Variations in Mechanical Properties of BZYx at Different Temperatures

**Figure 2:**
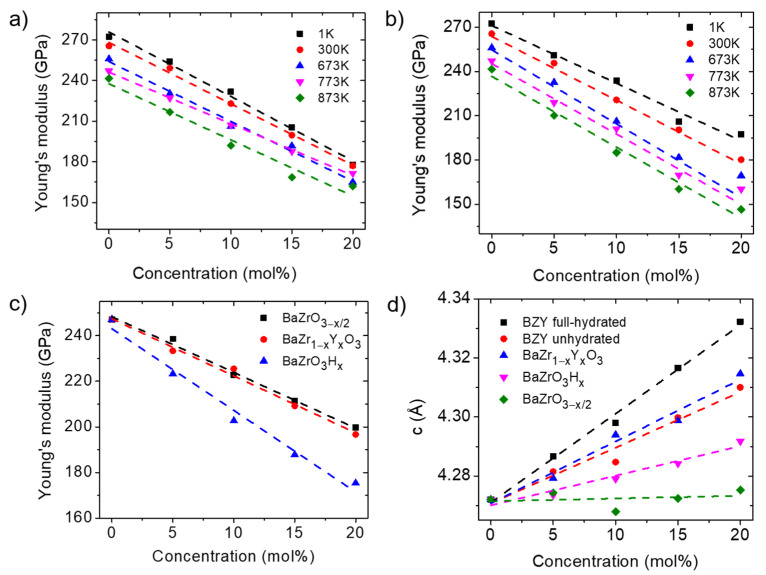
Analysis of the Young’s moduli of BZYx under different conditions. (**a**) The Young’s moduli of non-hydrated BZYx with different Y-doping concentration at different temperatures; (**b**) The Young’s moduli of full-hydrated BZYx with different Y-doping concentrations at different temperatures; (**c**) The Young’s moduli of BZYx with only Y doping, only oxygen vacancies, and only protonic point defects at 773 K; (**d**) Lattice constant of non-hydrated BZY, fully hydrated BZY, and BaZrO_3_ with only Y doping, only oxygen vacancies, and only protonic point defects at 773 K.

The Young’s moduli of fully hydrated and non-hydrated BZYx at different temperatures and doping concentrations are shown in Figure 2. The Young’s modulus of BaZrO_3_ at room temperature is calculated to be 265.58 GPa, which is consistent with Ref. [42]. Clearly, the Young’s modulus decreases with increasing temperature, regardless of the hydration state, as revealed by Figure 2a,b. The trend is consistent with the results of the experimental study that Young’s modulus of BZY decreased by 30–40% from room temperature to 773 K [42]. In addition, increasing the Y-doping concentration also decreases the Young’s modulus of BZYx in both hydrated and non-hydrated states. The ab initio calculations performed at room temperature by Hoedl et al. [43] also showed that with the increase in the concentration of Y doping, the Young’s modulus of both hydrated and non-hydrated BZY decreased. As revealed by this study, the trend is preserved at P-SOFC operating temperatures.

To further understand the impact of point defects on the Young’s moduli of BZYx, calculations were performed for cases with only doping and without oxygen vacancies (BaZr_1−*x*_Y*_x_*O_3_), only oxygen vacancies without Y doping (BaZrO_3−*x*/2_), and only protonic point defects (BaZrO_3_H*_x_*) at 773 K (*x* = 0.05, 0.1, 0.15, 0.2). The results are shown in Figure 2c. It can be seen that Y doping, oxygen vacancies, and protonic point defects all lead to a decrease in the Young’s modulus. Moreover, the lattice constants under these conditions at 773 K are shown in Figure 2d. Y doping and proton defects lead to an increase in the lattice constant. Among them, the effect of oxygen vacancies on the lattice constant is relatively small.

Based on the above results, the effects of point defects in BZYx on the Young’s moduli can be explained by the change in the interionic force and the chemical bond strength raised by the generation of the defects. First, the ionic radius of Y (0.9 Å) is larger than that of Zr (0.72 Å) [44]. Thus, doping with Y leads to an increase in the lattice constant, which reduces the interionic forces. Second, the substitution of Zr^4+^ with Y^3+^ decreases the cationic charge; the proton point defects replace the doubly charged oxygen ion with a singly charged hydroxide ion; the generation of oxygen vacancies reduces the number of metal–oxygen chemical bonds. All of these three factors contribute to the decrease in the chemical bond strength, which in turn leads to a decrease in the Young’s modulus [42,43].

### 3.2. Effects of Strains on the Proton Diffusion under Different Conditions

**Figure 3:**
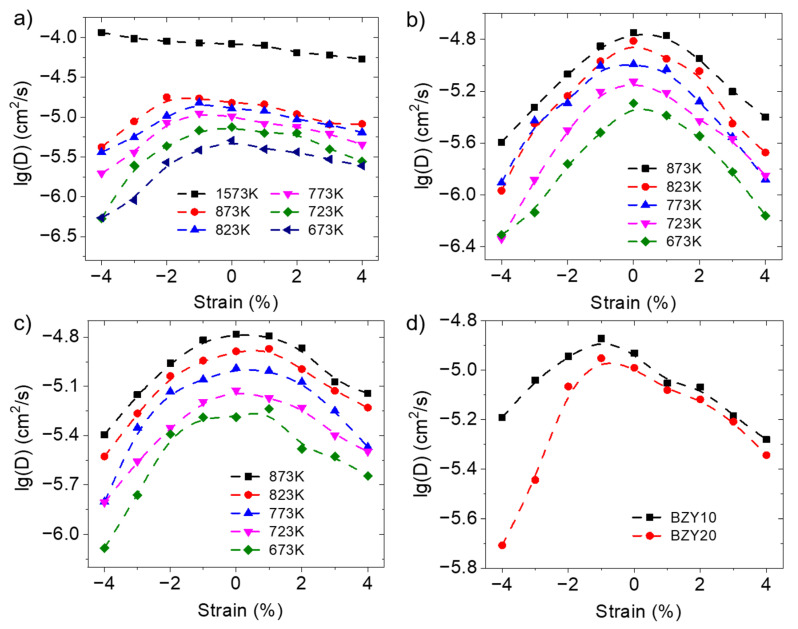
Diffusion coefficient of BZY10 and BZY20 under different conditions. (**a**) Diffusion coefficient of BZY20 at different temperatures and subject to isotropic strains; (**b**) Diffusion coefficient of BZY20 at different temperatures and subject to biaxial strains; (**c**) Diffusion coefficient of BZY20 at different temperatures and subject to uniaxial strains; (**d**) Diffusion coefficient of BZY10 and BZY20 subject to isotropic strains at 773 K.

Figure 3 shows the proton-diffusion coefficient of BZY20 at different temperatures and subject to different isotropic, biaxial, and uniaxial strains. For isotropic strain, the results are shown in Figure 3a. It can be seen that from 673 K to 873 K, the diffusion coefficient of the proton initially increases and then decreases as the system transitions from a compression state to a tension state. This result is not entirely consistent with the findings of Ottochian et al. [33], in which compression strain promotes proton conduction. Considering that the simulation temperature of their research was 1500 K–2000 K, we also conducted simulations at 1573 K and found that up to a compressive strain of 4%, compressive strain promotes proton diffusion, consistent with Ottochian’s results. We speculate that the promotion of proton rotation by the temperature increase attenuates the inhibitory effect of pressure strain on proton long-range diffusion, which will be discussed in detail in the next section. For uniaxial and biaxial strain (Figure 3b,c), the proton-diffusion coefficients similarly adhere to a trend of first increasing and then decreasing when transitioning from compressive to tensile strain. This is consistent with the findings of Ottochian et al. [34] and Fluri et al. [35] that showed a parabolic trend of the diffusion coefficient as a function of strain.

In addition, the proton-diffusion coefficients of BZY10 under different strains at 773 K are shown in Figure 3d and compared with BZY20. An increase in the Y-doping concentration leads to a decrease in the proton-diffusion coefficient. Furthermore, the lower Y-doping concentration enhances the inhibitory effect of pressure on proton diffusion. This could be a result of the higher lattice constant with the increase in Y-doping concentration [33]. In addition, proton trapping, which hinders proton transfer, could be another contributing factor, which will be discussed in the next section.

**Figure 4:**
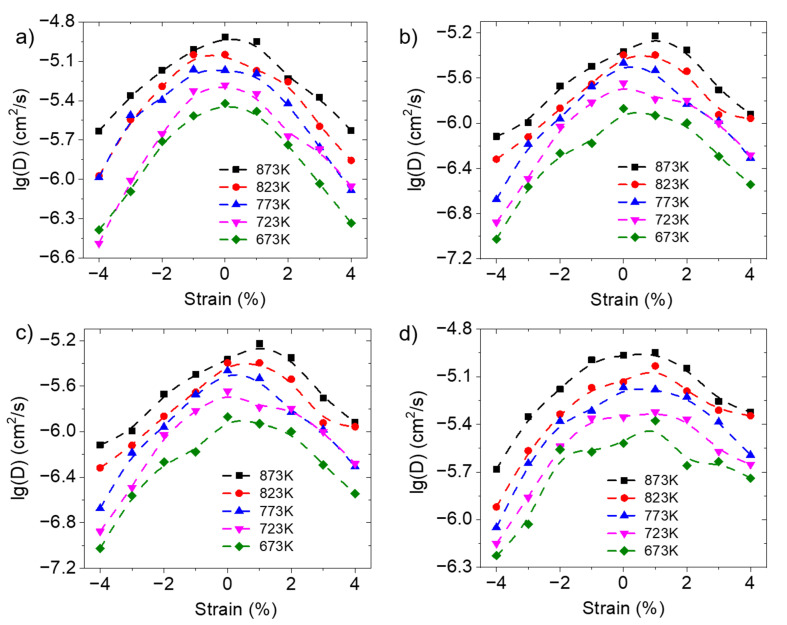
The proton-diffusion coefficients of BZY20 along and perpendicular to the deformation direction at different temperatures and strains. (**a**) Diffusion coefficients along the deformation direction under biaxial strain; (**b**) Diffusion coefficients perpendicular to the deformation direction under biaxial strain; (**c**) Diffusion coefficients along the deformation direction under uniaxial strain; (**d**) Diffusion coefficients perpendicular to the deformation direction under uniaxial strain.

When subject to anisotropic strain, it is important to investigate not only the overall diffusion rates but also the diffusion behavior of protons along and perpendicular to the plane of deformation. The proton-diffusion coefficients along the deformation direction and perpendicular to the deformation direction of BZY20 under biaxial and uniaxial strain are shown in Figure 4. The results also follow the trend of first increasing and then decreasing as the strain changes from compressive to tensile, the same as that of bulk diffusion. The analysis of the causes of this trend will be discussed in detail from the perspective of each elementary step of proton diffusion later.

### 3.3. Analysis of Factors Affecting Proton Diffusion under Different Strains

#### 3.3.1. Rotation of Hydroxide Ions

**Figure 5:**
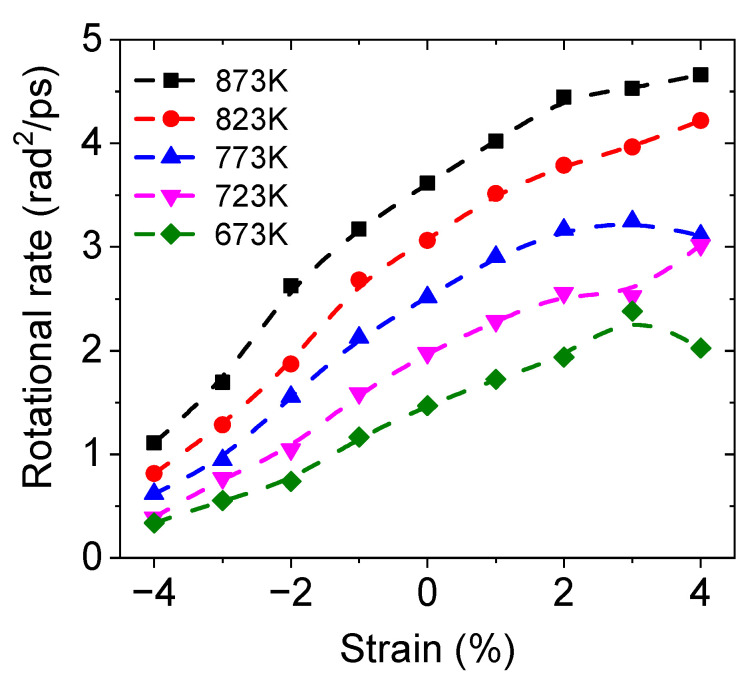
Rotational rate of BZY20 under different strains and temperatures.

The rotational rates, represented by the slopes of the MSAD curves, of protons in BZY20 under different temperatures and strains are shown in Figure 5. The rotational rate gradually increases with the strain changing from a compression state to a tension state, meaning that the compressive strain hinders the rotation of hydroxide ions. This trend is consistent with the result in Ref. [34] that compressive pressure increased the rotational energy barrier of hydroxide ions. Thus, the slower hydroxide rotation rate constitutes one of the factors accounting for the slower proton diffusion under higher compression strain.

#### 3.3.2. Protons Transfer between Oxygen Atoms

**Figure 6:**
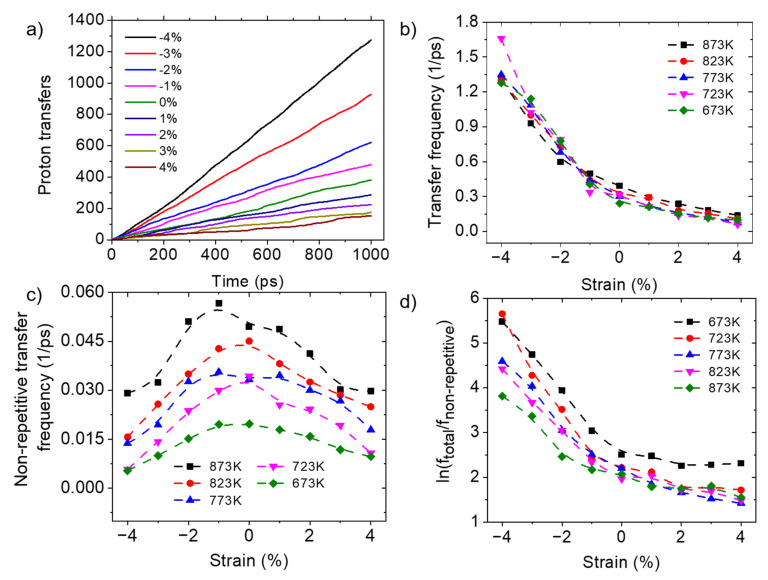
Proton transfers of BZY20 under different conditions. (**a**) The average total number of proton transfers changes over time under different strains at 873 K; (**b**) Total proton transfer frequency under different strains and temperatures; (**c**) Non-repetitive proton transfer frequency under different strains and temperatures; (**d**) Ratio of total and non-repetitive transfers under different strains and temperatures.

The average total number of transfers of all protons as a function of time at 873 K is shown in Figure 6a. The slope of this curve is defined here as the transfer frequency, with a unit of 1/ps. Figure 6b gives the dependence of the transfer frequencies on temperature and strain. The total number of transfers increases with increasing compressive strain, indicating that compressive strain can promote the transfer of protons between oxygen ions. Fronzi et al. [32] demonstrated that increasing compressive strain enhances the interaction between the proton and the adjacent oxygen atom to which the proton is transferred. This increased interaction facilitated the proton transfer to the next oxygen atom, thereby improving proton-transfer efficiency. Niu et al. [34] also showed that as the compressive strain increases, the energy barrier for proton transfer decreases. Overall, the effect of compressive strain on proton transfer counters the effect of compressive strain on the rotation of hydroxide ions. From another perspective, the suppressed proton transfer under tensile strains is one of the reasons for the reduced diffusion.

It should also be noted that not all of the proton transfers contribute to long-range proton diffusion. That is, only the protons which continue to transfer to the third O atom contribute to the long-range proton diffusion, while the protons transferring back to the original O atom do not. Thus, it is worthwhile to investigate the total number of proton transfers on non-repetitive oxygen sites. Figure 6c shows that the trend of the non-repetitive transfer frequency follows a similar trend of first increasing and then decreasing. Figure 6d gives the ratio between the total transfer and non-repetitive transfer. The higher ratio of repetitive transfer is because of the slower rotational rate of hydroxide on an O atom under compressive strain, under which condition the protons tend to transfer within a small region, thereby inhibiting the long-range transport of protons. Furthermore, as the temperature increases, the inhibition of proton long-range movement by compression weakens, as reflected by the lower ratio of the total transfer to non-repetitive transfer. This explains the discrepancies shown in Figure 3a that under compressive strains, proton transport is promoted at the higher temperature of 1573 K, while it is inhibited at lower temperatures of 673 K to 873 K.

#### 3.3.3. Proton Trapping

**Figure 7:**
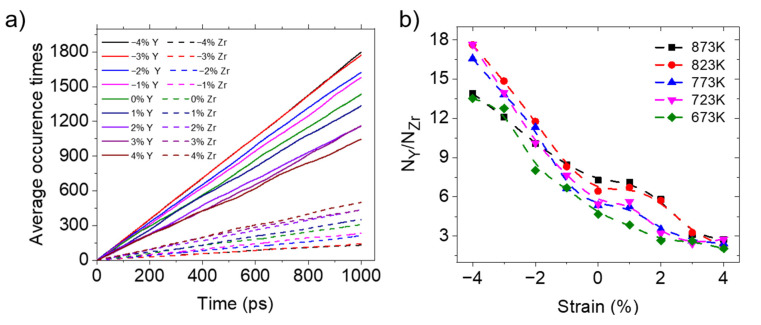
Effect of strains on the proton-trapping phenomenon in BZY20. (**a**) The average cumulative occurrences of H on O atoms centered around Y atoms and O atoms centered around Zr atoms under different strains at 873 K; (**b**) Ratio of the average cumulative occurrence probability of H on O atoms centered around Y atoms and O atoms centered around Zr atoms under different strains, at different temperatures.

The incorporation of lower-valence Y^3+^ ions causes the doping sites to carry equivalent negative charges [44]. These charged species, in combination with local strain effects, could trap protons and impede their long-range transport. Therefore, proton trapping is also an important factor affecting proton diffusion. In this section, the average frequencies of protons appearing on oxygen atoms centered around Y octahedral forms and those centered around Zr octahedral forms are counted and compared, in order to investigate the proton-trapping phenomenon by Y cations. Figure 7a gives the average cumulative occurrences of H atoms around the Zr atoms and Y atoms of BZY20 at 873 K with different strains. The results indicate that with increasing compressive strain, the cumulative occurrence of H atoms on O atoms centered around Y atoms increases, while the occurrence on O atoms centered around Zr atoms decreases. The extent of proton trapping is assessed by the ratio of the average cumulative occurrence slope of protons appearing on oxygen atoms surrounding Y to those surrounding Zr, and the result is shown in Figure 7b. This suggests that under compressive strain, it is more difficult for protons to escape the trapping. According to the research of Fluri et al. [35], the increased distance between oxide ions and dopants reduces the association energy between protons and dopants, which facilitates the escape of protons from dopant sites. Therefore, proton trapping is diminished under tensile conditions. Proton trapping is also one of the reasons for the higher proton-diffusion coefficient of BZY10 than BZY20, due to the lower concentration of Y doping, while the apparent low conductivity of BZY10 is due to the lower total number of protons.

#### 3.3.4. Impact of Anisotropic Strain on Proton Distribution

**Figure 8:**
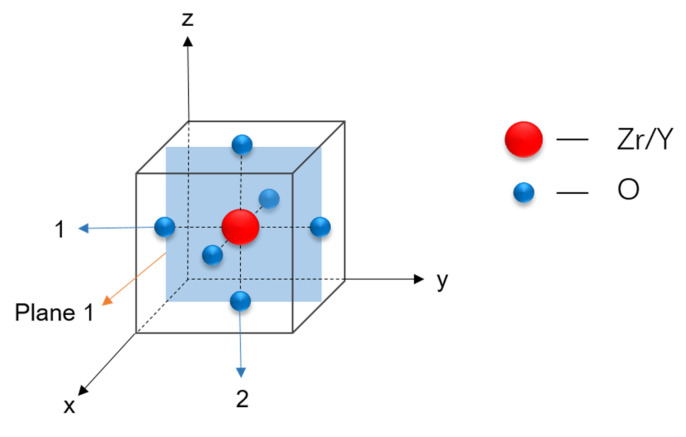
Schematic diagram of a unit Zr/Y-centered octahedral, with biaxial strain applied along the *xy*-directions. Plane 1: Zr(Y)O_2_ plane perpendicular to the strain plane; Oxygen atoms 1: Oxygen atoms distributed along the strain direction; Oxygen atoms 2: Oxygen atoms distributed perpendicular to the strain direction.

**Figure 9:**
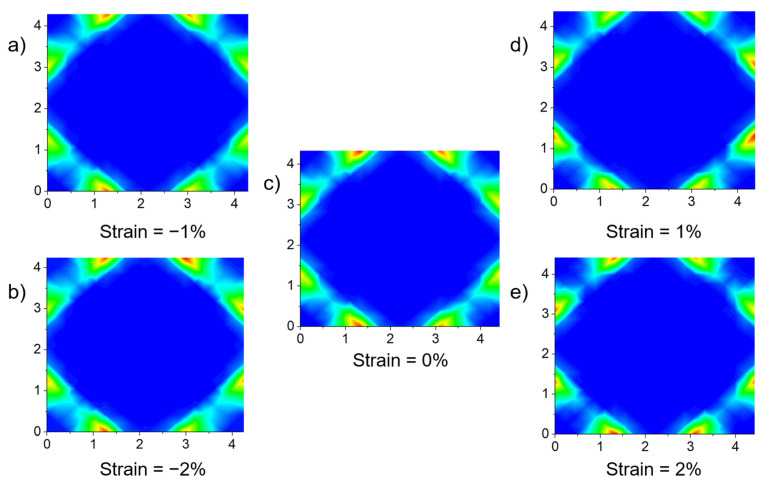
Proton trajectories of BZY20 in Plane 1 under different strains at 873 K.

Figure 8 gives an illustration of a unit Zr/Y-centered octahedral. When biaxial strain is applied in the *xy*-directions, Plane 1—shown in Figure 8—is the Zr(Y)O_2_ plane perpendicular to the direction of the strain. To understand the trajectories of protons under different deformation conditions, the occurrence frequency of protons on Plane 1 was calculated, and the resulted proton density is illustrated in Figure 9. In the absence of applied strain (Figure 9c), the proton distribution is uniform. With an increase in biaxial tensile strain, the proton distribution becomes more concentrated in the *y*-direction and decreases in the *z*-direction. Conversely, with the increase in biaxial compressive strain, the proton distribution becomes more concentrated in the *z*-direction and decreases in the *y*-direction.

**Figure 10:**
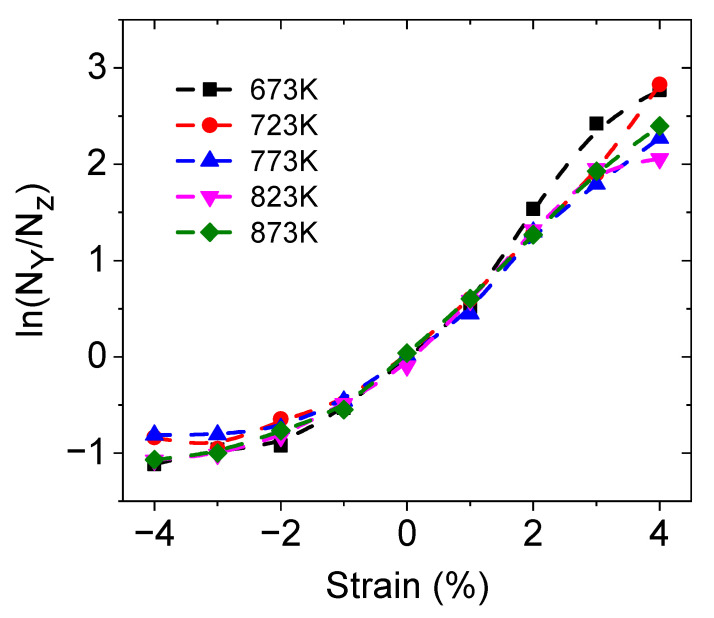
Ratio of the average cumulative occurrence probability of H on O along the *y*-direction (N*_y_*) and *z*-direction (N*_z_*) under different strains and at different temperatures.

Additionally, to enable quantitative evaluations, the O atoms in an octahedral form are divided into two categories: the first category is distributed along the *y*-direction (the direction of strain), and the second category is distributed along the *z*-direction (perpendicular to the direction of strain), labeled as 1 and 2, respectively, in Figure 8. The total occurrences of protons on these two types of oxygen ions were counted over time, and the ratios of their respective slopes were calculated, which are shown in Figure 10. These results also indicate that protons preferentially occupy the O atoms with greater spatial availability, which is consistent with the research of Ottochian et al. [33]. This means that in the direction of tension, the number of protons increases, but tension inhibits proton diffusion. The proton distribution, along with the three previous factors, affects proton diffusion under anisotropic strains.

## 4. Conclusions

In this study, the mechanical properties and the variation in proton conductivity of BZY under isotropic and anisotropic strains at elevated temperatures are investigated. Moreover, this research distinguishes itself by offering a comprehensive analysis of the effects of strain on key factors influencing proton diffusion, including hydroxide rotation, proton transfer, proton trapping, and proton distribution—an aspect that has not been thoroughly addressed in previous studies. For both hydrated and non-hydrated BZY, the Young’s modulus increases with an increasing doping concentration of Y. This is a cumulative result of Y doping, the generation of oxygen vacancies, and the generation of proton defects, all of which decrease the Young’s modulus of BZY by weakening the strength of chemical bonds. From 673 K to 873 K, the conductivity follows the rule of first increasing and then decreasing with the strain transitioning from a compressive state to a tensile state under different conditions. It is found that isotropic compressive strain promotes proton transfer but inhibits hydroxide ion rotation and exacerbates proton trapping. For anisotropic strain, in addition to these three factors, protons tend to be distributed on oxygen atoms with a larger space. This promotes diffusion in the tensile direction and inhibits diffusion in the compressive direction. The effect of strain on these factors determines proton conduction collectively.

## Data Availability

The original contributions presented in the study are included in the article, further inquiries can be directed to the corresponding authors.

## References

[1] Atkinson A., Barnett S., Gorte R.J., Irvine J.T.S., McEvoy A.J., Mogensen M., Singhal S.C., Vohs J. (2004). Advanced anodes for high-temperature fuel cells. Nat. Mater..

[2] Sazali N. (2020). Emerging technologies by hydrogen: A review. Int. J. Hydrogen Energy.

[3] Pan Z., Shen J., Wang J., Xu X., Chan W.P., Liu S., Zhou Y., Yan Z., Jiao Z., Lim T.-T. (2022). Thermodynamic analyses of a standalone diesel-fueled distributed power generation system based on solid oxide fuel cells. Appl. Energy.

[4] Quach T.-Q., Lee D., Giap V.-T., Kim Y.S., Lee S., Ahn K.Y. (2024). Energetic and economic analysis of novel cascade systems for ammonia-fed solid oxide fuel cell. Int. J. Hydrogen Energy.

[5] Yang W., Pan Z., Jiao Z., Zhong Z., O’Hayre R. (2024). Advanced microstructure characterization and microstructural evolution of porous cermet electrodes in solid oxide cells: A comprehensive review. Energy Rev..

[6] Vinchhi P., Khandla M., Chaudhary K., Pati R. (2023). Recent advances on electrolyte materials for SOFC: A review. Inorg. Chem. Commun..

[7] Lee T.H., Kim K.D., Jung U., Im H.B., Koo K.Y. (2023). Evaluation of monolith catalyst in catalytic combustion of anode off-gas for solid oxide fuel cell system. Catal. Today.

[8] Razmi A.R., Sharifi S., Vafaeenezhad S., Hanifi A.R., Shahbakhti M. (2024). Modeling and microstructural study of anode-supported solid oxide fuel cells: Experimental and thermodynamic analyses. Int. J. Hydrogen Energy.

[9] Leng Y., Chan S.H., Liu Q. (2008). Development of LSCF–GDC composite cathodes for low-temperature solid oxide fuel cells with thin film GDC electrolyte. Int. J. Hydrogen Energy.

[10] Leng Y.J., Chan S.H., Jiang S.P., Khor K.A. (2004). Low-temperature SOFC with thin film GDC electrolyte prepared in situ by solid-state reaction. Solid State Ion..

[11] Lin C., Zhang Y., Qian J., Chen Z., Huang J., Ai N., Jiang S.P., Wang X., Shao Y., Chen K. (2024). Coupling of tape casting and in situ solid-state reaction for manufacturing La_0.9_Sr_0.1_Ga_0.8_Mg_0.2_O_3_ electrolyte of efficient solid oxide cells. J. Eur. Ceram. Soc..

[12] Shah M.A.K.Y., Lu Y., Mushtaq N., Yousaf M., Lund P.D., Asghar M.I., Zhu B. (2023). Designing Gadolinium-doped ceria electrolyte for low temperature electrochemical energy conversion. International Journal of Hydrogen Energy.

[13] Kreuer K.D. (2003). Proton-Conducting Oxides. Annu. Rev..

[14] Liang M., He F., Zhou C., Chen Y., Ran R., Yang G., Zhou W., Shao Z. (2021). Nickel-doped BaCo_0.4_Fe_0.4_Zr_0.1_Y_0.1_O_3−δ_ as a new high-performance cathode for both oxygen-ion and proton conducting fuel cells. Chem. Eng. J..

[15] Peng S., Lei S., Wen S., Weng G., Ouyang K., Yin Z., Xue J. (2023). High-performance solid oxide fuel cell based on iron and tantalum co-doped BaCoO_3−δ_ perovskite-type cathode and BaZr_0.1_Ce_0.7_Y_0.1_Yb_0.1_O_3−δ_ electrolyte. Int. J. Hydrogen Energy.

[16] Lin C.-K., Chen T.-T., Chyou Y.-P., Chiang L.-K. (2007). Thermal stress analysis of a planar SOFC stack. J. Power Sources.

[17] Xu M., Li T.S., Yang M., Andersson M., Fransson I., Larsson T., Sundén B. (2016). Modeling of an anode supported solid oxide fuel cell focusing on thermal stresses. Int. J. Hydrogen Energy.

[18] Yakabe H., Baba Y., Sakurai T., Yoshitaka Y. (2004). Evaluation of the residual stress for anode-supported SOFCs. J. Power Sources.

[19] Wang Y., Jiang W., Luo Y., Song M. (2022). High temperature creep strength design and optimization of solid oxide fuel cell. Int. J. Hydrogen Energy.

[20] Da Y., Xiao Y., Zhong Z., Pan Z., Jiao Z. (2022). Predictions on conductivity and mechanical property evolutions of yttria-stabilized zirconia in solid oxide fuel cells based on phase-field modeling of cubic-tetragonal phase transformation. J. Eur. Ceram. Soc..

[21] Wang Y., Jiang W., Song M., Luo Y., Tu S.-T. (2020). Effect of inhomogeneous oxidation on the mechanical degradation of anode supported solid oxide fuel cell. J. Power Sources.

[22] Wang Y., Jiang W., Luo Y., Zhang Y., Tu S.-T. (2017). Evolution of thermal stress and failure probability during reduction and re-oxidation of solid oxide fuel cell. J. Power Sources.

[23] Kim Y.J., Lee M.C. (2017). Numerical investigation of flow/heat transfer and structural stress in a planar solid oxide fuel cell. Int. J. Hydrogen Energy.

[24] Zhang Q., Xie K., Luo Y., Zhang Y.-C., Jiang W.-C. (2022). Mismatch effect of material creep strength on creep damage and failure probability of planar solid oxide fuel cell. Int. J. Hydrogen Energy.

[25] Sato K., Suzuki K., Narumi R., Yashiro K., Hashida T., Mizusaki J. (2011). Ionic Conductivity in Uniaxial Micro Strain/Stress Fields of Yttria-Stabilized Zirconia. Jpn. J. Appl. Phys..

[26] Shen W., Jiang J., Hertz J.L. (2014). Reduced ionic conductivity in biaxially compressed ceria. RSC Adv..

[27] Fluri A., Pergolesi D., Roddatis V., Wokaun A., Lippert T. (2016). In situ stress observation in oxide films and how tensile stress influences oxygen ion conduction. Nat Commun.

[28] Goodenough J.B. (2003). Oxide-Ion Electrolytes. Annu. Rev..

[29] Zhu H., Ricote S., Kee R.J. (2022). Faradaic efficiency in protonic-ceramic electrolysis cells. J. Phys. Energy.

[30] Chen Q., Braun A., Yoon S., Bagdassarov N., Graule T. (2011). Effect of lattice volume and compressive strain on the conductivity of BaCeY-oxide ceramic proton conductors. J. Eur. Ceram. Soc..

[31] Braun A., Ovalle A., Pomjakushin V.Y., Cervellino A., Erat S., Stolte W.C., Graule T.J.A.P.L. (2009). Yttrium and hydrogen superstructure and correlation of lattice expansion and proton conductivity in the BaZr_0.9_Y_0.1_O_2.95_ proton conductor. Appl. Phys. Lett..

[32] Fronzi M., Tateyama Y., Marzari N., Nolan M., Traversa E. (2016). First-principles molecular dynamics simulations of proton diffusion in cubic BaZrO_3_ perovskite under strain conditions. Mater. Renew. Sustain. Energy.

[33] Ottochian A., Dezanneau G., Gilles C., Raiteri P., Knight C., Gale J.D. (2014). Influence of isotropic and biaxial strain on proton conduction in Y-doped BaZrO_3_: A reactive molecular dynamics study. J. Mater. Chem. A.

[34] Niu H., Jing Y., Sun Y., Guo L., Aluru N.R., Li W., Yang J., Li X. (2022). Strain-induced tunable energy barrier of proton diffusion in Y-doped BaCeO_3_ and Y-doped BaZrO_3_. Int. J. Energy Res..

[35] Fluri A., Marcolongo A., Roddatis V., Wokaun A., Pergolesi D., Marzari N., Lippert T. (2017). Enhanced Proton Conductivity in Y-Doped BaZrO_3_ via Strain Engineering. Adv. Sci..

[36] Niu H., Jing Y., Sun Y., Guo L., Aluru N.R., Li W., Yang J., Li X. (2022). On the anomalous diffusion of proton in Y-doped BaZrO_3_ perovskite oxide. Solid State Ion..

[37] Thompson A.P., Aktulga H.M., Berger R., Bolintineanu D.S., Brown W.M., Crozier P.S., In’t Veld P.J., Kohlmeyer A., Moore S.G., Nguyen T.D. (2022). LAMMPS—A flexible simulation tool for particle-based materials modeling at the atomic, meso, and continuum scales. Comput. Phys. Commun..

[38] Stokes S.J., Islam M.S. (2010). Defect chemistry and proton-dopant association in BaZrO_3_ and BaPrO_3_. J. Mater. Chem..

[39] Araki W., Arai Y. (2010). Molecular dynamics study on oxygen diffusion in yttria-stabilized zirconia subjected to uniaxial stress in terms of yttria concentration and stress direction. Solid State Ion..

[40] Edmond K.V., Elsesser M.T., Hunter G.L., Pine D.J., Weeks E.R. (2012). Decoupling of rotational and translational diffusion in supercooled colloidal fluids. Proc. Natl. Acad. Sci. USA.

[41] Iguchi F., Hinata K. (2021). High-Temperature Elastic Properties of Yttrium-Doped Barium Zirconate. Metals.

[42] Hoedl M.F., Makagon E., Lubomirsky I., Merkle R., Kotomin E.A., Maier J. (2018). Impact of point defects on the elastic properties of BaZrO_3_: Comprehensive insight from experiments and ab initio calculations. Acta Mater..

[43] Makagon E., Merkle R., Maier J., Lubomirsky I. (2020). Influence of hydration and dopant ionic radius on the elastic properties of BaZrO_3_. Solid State Ion..

[44] Yamazaki Y., Blanc F., Okuyama Y., Buannic L., Lucio-Vega J.C., Grey C.P., Haile S.M. (2013). Proton trapping in yttrium-doped barium zirconate. Nat. Mater..

